# Didn’t inhale? Time to reconsider aerosolized antibiotics in the treatment of ventilator-associated pneumonia

**DOI:** 10.1186/s13054-018-2205-8

**Published:** 2018-12-05

**Authors:** Daniel A. Sweeney, Andre C. Kalil

**Affiliations:** 1 Division of Pulmonary, Critical Care and Sleep Medicine, University of California, San Diego, CA USA; 2 Division of Infectious Diseases, Department of Medicine, University of Nebraska Medical Center, 985400 Nebraska Medical Center, Omaha, NE 68135 USA

**Keywords:** Inhaled, Aerosolized, Antibiotics, Ventilator-associated pneumonia

“The War on Drugs” in the 1970s had an unintended effect on the illegal cannabis industry, increasing domestic production such that cannabis has become among the most profitable cash crops in the United States [1]. Legal barriers to cannabis use are also steadily crumbling: 30 states have legalized medical marijuana and nine states permit recreational use [2]. Yet despite its economic impact and increasing mainstream use, the safety and merits of cannabis consumption have not been rigorously studied. Is cannabis an effective antiepileptic or does it cause seizures? How is it possible that cannabis is touted as an anti-emetic, yet cannabinoid hyperemesis syndrome is reported in the medical literature? The answers to these questions likely relate to dose, mode of delivery, and patient selection—the very same questions at the root of whether inhaled antibiotics are an effective therapy for ventilator-associated pneumonia (VAP).

VAP, defined as pneumonia developing 48 h after intubation, is associated with increased intensive care unit (ICU) stay and duration of mechanical ventilation and may independently impact mortality [3, 4]. The burden of VAP on the healthcare system is not trivial, with survey data suggesting that VAP represents close to 10% of all hospital-acquired infections [5]. The problem of VAP is more complex in light of the growing scourge of multidrug resistant (MDR) organisms. Coupled with a paucity of new antibiotics, clinicians and researchers have turned their attention to improving the delivery of tried-and-true medications, including the use of extended intravenous effusions and aerosolized antibiotics. Inhaled antimicrobials have been used since the 1940s to treat various respiratory infections. Yet, only three aerosolized antibiotics (aztreonam, tobramycin, and colistin) have received either FDA or European Medicines approval and only for the treatment of infections in patients with cystic fibrosis [6].

The role of inhaled antibiotics for VAP has been studied in numerous independent small randomized trials enrolling—in sum—approximately 400 patients and described in various observational studies totaling nearly 700 patients. These studies encompass heterogeneous populations, infected with different MDR organisms, treated with various antibiotics administered as either a solo or adjunctive therapy, delivered via a variety of technologies (e.g., jet nebulizer, ultrasonic nebulizer, vibrating mesh nebulizer), and used different outcome endpoints [7, 8]. Thus, interpreting these results is a challenging task. Alternatively, it is remarkable that most randomized or observational studies of inhaled antibiotics for VAP have shown some potential benefit (either mortality, clinical recovery, or microbiologic clearance) and low risk for harms such as systemic antibiotic toxicity or development of new antimicrobial resistance. In fact, the high peak concentration and the low systemic exposure of inhaled antibiotics may lead to less selective pressure and lower development to bacterial resistance than intravenous antibiotics.

In light of the challenges to interpret the current evidence on the efficacy of inhaled antibiotics, Xu et al. [9] performed both a standard and a network meta-analysis (NMA) involving randomized and observational studies to expand our understanding of the effect of inhaled antibiotics for the treatment of VAP. The findings from the standard meta-analysis suggest significant benefits from the adjunctive use of inhaled antibiotics compared to intravenous therapies alone in terms of both clinical recovery and microbiological eradication; adjunctive aerosolized antibiotic therapy conferred no differences regarding mortality and nephrotoxicity outcomes. Alternatively, the results of the NMA suggest that clinical recovery benefits, microbiological eradication, and survival were each associated with different aerosolized antibiotics. Hence, this comprehensive analysis suggests a potential benefit of inhaled antibiotics for patients with VAP, but not to a degree that would warrant clinicians deviating from the current American Thoracic Society (ATS)/Infectious Diseases Society of America (IDSA) recommendation that the use of both inhaled and systemic antibiotics should only be considered when the bacterial etiology of the pneumonia is due to MDR Gram-negative microorganisms sensitive only to aminoglycosides or polymyxins [3].

A coordinated effort to study inhaled antibiotics for the treatment of VAP is needed. And future investigations must pay particular attention to specific variables and study design characteristics. Patient selection is extremely important. There is no gold standard definition for VAP, and many prior studies of ICU patients with suspected pneumonia have been limited by the inclusion of patients who were not infected. Including objective measures such as hypoxia, severity scores, biomarkers, and rapid PCR diagnostics when defining the trial population could reduce this risk. The impact of the drug delivery systems also needs to be studied and should include currently available and inexpensive delivery systems such a jet nebulization so as to increase the chances of finding a broad reaching beneficial intervention. Finally, future trials should insist on hard and meaningful endpoints such as time on mechanical ventilation, ICU days, and mortality. Thus, the current body of evidence suggests that the case for inhaled antibiotics in VAP may have great potential and is worthy of study. But like the controversy surrounding medical and recreational marijuana use, clarity on the issue of inhaled antibiotics for VAP will only be achieved with prospective studies that take into consideration patient characteristics, drug dose, and mode of delivery and utilize objective endpoints.
